# Ghana Heart Initiative Training for Cardiac Arrest Management Among Health Care Professionals: Outcomes Evaluation Study (2019-2024)

**DOI:** 10.2196/75536

**Published:** 2025-12-31

**Authors:** Alfred Doku, Lawrence Sena Tuglo, Chiedozie Osuoji, Juliette Edzeame, Marisa Broni, David Danso Mainoo, Alberta Ewuziwaa Acquah, Kwatetso Honny, Ron J G Peters, Charles Agyemang

**Affiliations:** 1 Department of Medicine and Therapeutics University of Ghana Medical School University of Ghana Accra Ghana; 2 Department of Public & Occupational Health University of Amsterdam Medical Centre University of Amsterdam Amsterdam, Netherlands The Netherlands; 3 Department of Nutrition and Dietetics School of Allied Health Sciences University of Health and Allied Sciences Ho Ghana; 4 Code Red Emergency Medical Services Accra Ghana; 5 Aya Integrated Healthcare Initiative International Services Deutsche Gesellschaft fur Internationale Zusammenarbeit (GIZ) Accra Ghana; 6 Department of Cardiology Amsterdam University Medical Centers University of Amsterdam Amsterdam The Netherlands; 7 Department of Medicine Johns Hopkins University School of Medicine Johns Hopkins University Baltimore, MD United States

**Keywords:** knowledge, skill assessment, basic life support, advanced cardiac life support, health professionals, Ghana

## Abstract

**Background:**

Health care professionals must stay updated with the latest guidelines for basic life support (BLS) and advanced cardiac life support (ACLS) to effectively assist patients during cardiac emergencies. Since its launch in 2018, the Ghana Heart Initiative has significantly enhanced the skills and knowledge of health care professionals in managing cardiovascular diseases, including cardiac emergencies.

**Objective:**

This study aims to assess the knowledge and skills of BLS and ACLS among health care professionals immediately after training in Ghana.

**Methods:**

This cross-sectional, training-based study involved 541 and 302 health care professionals trained in BLS and ACLS, respectively. Among them, 229 BLS and 124 ACLS-trained participants completed the questionnaires immediately after the training, and their data were included in the final analysis. Knowledge was assessed using a standardized questionnaire and an instructor-led skills evaluation based on the updated 2018 and 2020 American Heart Association guidelines for cardiopulmonary resuscitation and emergency cardiovascular care.

**Results:**

This study shows that 74.6% (171/229) of the health care professionals had adequate knowledge and skills in BLS. Those working in tertiary health care facilities were 80% less likely (adjusted odds ratio [AOR] 0.20, 95% CI 0.07-0.59; *P*=.003) to have adequate BLS knowledge and skills than those in primary health care facilities. Health care professionals from regions such as Volta and Oti were 4.94 times more likely to have adequate BLS knowledge and skills compared to those from Bono East (AOR 4.94, 95% CI 1.17-20.80; *P*=.03). Over 73.3% (91/124) of health care professionals had adequate knowledge and skills in ACLS. Males were 7.05 times more likely (AOR 7.05, 95% CI 2.69-18.46; *P*<.001) than females to possess adequate ACLS knowledge and skills.

**Conclusions:**

Given an opportunity to learn and practice, health care professionals in Ghana attain adequate knowledge and skills in BLS and ACLS.

## Introduction

Cardiac arrest, the sudden and complete shutdown of heart function, causes both respiratory and circulatory failure [1]. It is a critical emergency that demands immediate action [1]. Most adult cardiac arrests occur suddenly mainly due to heart diseases or systemic issues such as electrolyte and acid-base imbalances [2]. Therefore, delivering chest compression is essential for maintaining blood flow [3]. Prompt initiation of cardiopulmonary resuscitation (CPR) is crucial because it temporarily preserves life by ensuring that sufficient blood reaches vital organs such as the brain and heart [4]. Knowledge and skills in basic life support (BLS) and advanced cardiac life support (ACLS) are vital for managing sudden cardiac arrest, myocardial infarction, foreign body airway obstructions, respiratory failure [3], and other serious cardiovascular diseases (CVDs) [5,6].

The American Heart Association (AHA) plays a crucial role in emergency cardiovascular care (ECC) by regularly updating the CPR and ECC guidelines [7,8]. These guidelines provide a standard approach for delivering BLS and ACLS to revive patients [7,8]. BLS, which involves a series of care given to patients experiencing respiratory arrest, cardiac arrest, or airway obstruction, is a fundamental part of these guidelines [9]. The BLS course, based on AHA guidelines, teaches participants how to quickly recognize life-threatening emergencies, perform effective chest compressions, provide proper ventilation, and use an automated external defibrillator [9]. Similarly, the ACLS, also based on AHA guidelines, includes recognizing signs of sudden cardiac arrest, myocardial infarction, and complete airway obstruction, as well as performing CPR and defibrillation with an automated external defibrillator [1]. Following these guidelines, ACLS and CPR, when administered promptly, have been shown to improve survival rates in medical emergencies [1].

As health care professionals play a vital role in emergency response, they are expected to possess a strong understanding of the basic resuscitation principles in patient care and stay updated with the BLS and ACLS guidelines for rescuing patients who are unconscious or in cardiac arrest. However, studies conducted in low-income countries such as South Africa [10], Afghanistan [2], and Pakistan [11] have reported limited BLS knowledge and skills among health care professionals. Additionally, research in Ethiopia [1] and Spain [4] has shown insufficient understanding of ACLS among health care professionals (nurses and doctors) and nurses, respectively. Unlike in high-income countries, where strict licensing or certification is required before practice, few studies [1,2,9,11,12] have evaluated the BLS and ACLS knowledge of health care professionals in low-income countries.

In Ghana, there are no strict licensing requirements for health care professionals to be trained in current BLS and ACLS guidelines or to obtain a certificate before practicing. However, through the Ghana Heart Initiative, some health care professionals in Ghana have received training in BLS and ACLS based on AHA-certified guidelines, filling this critical gap in the health system [13]. To our knowledge, no study has assessed the knowledge and skills in BLS and ACLS among health care professionals in Ghana. Therefore, this study aims to evaluate their knowledge and skills in BLS and ACLS through training, enabling them to manage cardiac arrest effectively in Ghana.

## Methods

### Study Setting and Recruitment

This study was conducted from 2019 to 2024 across 8 of Ghana’s 16 administrative regions. These include Ashanti, Bono East, Central, Eastern, Greater Accra, Northern, Volta, and Oti. Ghana, located in West Africa, is classified as a lower-middle-income country. Ghana borders the Gulf of Guinea and the Atlantic Ocean, sharing borders with Côte d'Ivoire to the west, Burkina Faso to the north, and Togo to the east [14]. As of 2021, Ghana had an estimated population of 33 million, covering approximately 238,535 square kilometers. Accra serves as the capital and functions as the country's economic and administrative center [14]. To ensure fair representation, proportional sampling was used to select 44 health facilities, including primary, secondary, and tertiary levels, as well as both public and quasi-governmental facilities. These included 6 health centers, 27 secondary facilities, 5 tertiary hospitals, and 6 community health planning and services facilities [15]. Written memos were sent to the administrators of the selected health facilities, requesting at least 1 health professional such as a physician assistant, nurse, house officer, medical officer, senior medical officer, specialist, or trainee specialist to participate in the training. The sessions took place in the capital cities of these regions at designated locations to encourage high attendance among health care professionals.

### Study Design and Population

**Figure 1:**
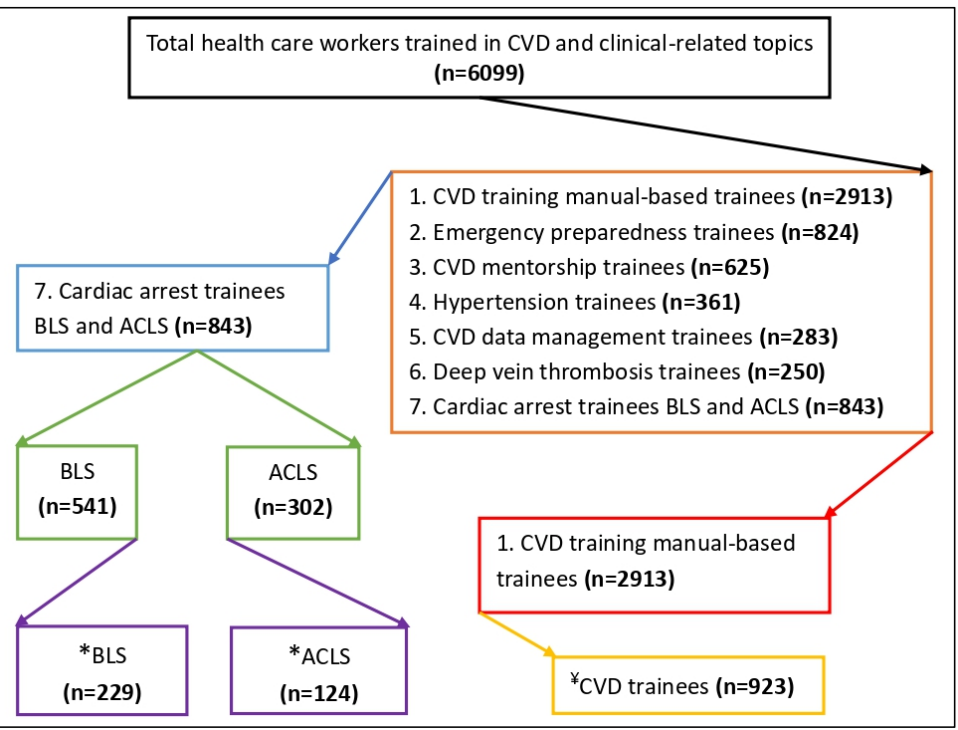
Flow diagram of health care professionals trained in CVDs and clinical topics. *Completed the BLS and ACLS training questionnaires and included in the final analysis. ¥The details of CVD trainees and the data are presented in a different study. ACLS: advanced cardiac life support; BLS: basic life support; CVD: cardiovascular disease.

This study employs a cross-sectional, training-based design involving 541 and 302 health care professionals recruited from selected health facilities and trained in BLS and ACLS, respectively. Among them, 229 BLS and 124 ACLS-trained participants completed the questionnaires immediately after the training, and their data were included in the final analysis (Figure 1).

### BLS and ACLS training

BLS and ACLS training took place over 1 and 3 days, respectively, conducted by Code Red, a certified training provider experienced in AHA. The course guide was based on the updated 2018 [7] and 2020 [8] AHA guidelines for CPR and ECC and was provided to participants at least 4 weeks prior to the training for preparation. The training packages included lectures with PowerPoint presentations, video materials, practical demonstrations, and hands-on exercises. The BLS course covered, among other topics, the chain of survival, the use of automated external defibrillators, team dynamics, CPR for infants and children, ventilation techniques, opioid-related emergencies, and choking. For ACLS, topics included, among others, cardiac arrest, acute coronary syndromes, stroke, arrhythmias, respiratory arrest, types of cardiac arrest (pulseless electrical activity, asystole, and ventricular tachycardia/fibrillation), and postcardiac arrest care.

### Data Collection and Assessment

After the training, an assessment of knowledge was conducted immediately through the self-administration of a standardized questionnaire and CPR skills drills, according to the updated 2018 [7] and 2020 [8] AHA guidelines for CPR and ECC. The questionnaire included demographics (gender, profession, facility type, and region) as well as 20 multiple-choice questions on BLS and ACLS. Ten questions were on BLS, and the other 10 questions were on ACLS. The participants took at least 30-45 minutes immediately after the training to complete the questionnaire.

### Classification of Knowledge and Skill Scores for BLS and ACLS

The knowledge and skill scores on BLS and ACLS were categorized into 2 levels. Scores ≥90% are considered adequate, while scores <90% are regarded as inadequate.

### Statistical Analysis

Data were coded and entered into Microsoft Excel 2016. Data were imported into SPSS software (version 25; IBM Corp) for analysis. Categorical data were presented as frequencies and percentages. When appropriate, the chi-square test or Fisher exact test was used to determine the associations between the BLS and ACLS knowledge and skill scores and demographic data. A *P* value <.05 was considered significant, and only those variables were included in the logistic regression analyses to test for associations.

### Ethical Considerations

Ethical clearance was obtained from the Ghana Health Service Ethics Review Committee (approval 018/05/19). Informed consent was obtained from all participants after the purpose of the training was explained to them in English. Data from participants who opted out at any time from the study or incomplete data were not included in the final analysis. Privacy and confidentiality were ensured by completing the questionnaires individually, and the data were used following their consent. Participants were compensated (money for transportation and attendance and food such as breakfast, lunch, snacks, and take-home supper) for their participation in the training based on the number of days they participated. Our study adhered to the principles of the Declaration of Helsinki.

## Results

### BLS Training

A total of 229 health care professionals received BLS training, and immediately standardized questionnaires were administered for the assessment of knowledge and skills. The majority (152/229, 66.3%) were females. Most (107/229, 46.7%) were specialists or trainee specialists, with 48.1% (110/229) working in secondary health care facilities. Additionally, 26.2% (60/229) of the BLS training sessions were held in the Greater Accra region. The majority (171/229, 74.6%) demonstrated adequate knowledge and skills in BLS. We identified statistically significant associations between profession, facility, region, and BLS knowledge and skill scores (*P*<.05; Table 1).

**Table 1:** Sociodemographic characteristics of the respondents receiving basic life support training.

Variable			Total (N=229), n (%)		Basic life support knowledge score					*P* value	
					Adequate (n=171), n (%)		Inadequate (n=58), n (%)				
**Gender**											.42
	Female	152 (66.4)		111 (64.9)		41 (70.7)					
	Male	77 (33.6)		60 (35.1)		17 (29.3)					
**Profession**											.03^a^
	Physician assistant/nurse	86 (37.6)		57 (33.3)		29 (50)					
	HO/MO/SMO^b^	36 (15.7)		32 (18.7)		4 (6.9)					
	Specialist/trainee specialist	107 (46.7)		82 (48)		25 (43.1)					
**Facility**											.008^c^
	Primary	64 (27.9)		57 (33.3)		7 (12.1)					
	Secondary	110 (48)		76 (44.4)		34 (58.6)					
	Tertiary	55 (24)		38 (22.2)		17 (29.3)					
**Region**											.03^a^
	Ashanti	29 (12.7)		22 (12.9)		7 (12.1)					
	Bono East	25 (10.9)		12 (7)		13 (22.4)					
	Central	25 (10.9)		19 (11.1)		6 (10.3)					
	Eastern	23 (10)		16 (9.4)		7 (12.1)					
	Greater Accra	60 (26.2)		49 (28.7)		11 (19)					
	Northern	32 (14)		23 (13.5)		9 (15.5)					
	Volta/Oti	35 (15.3)		30 (17.5)		5 (8.6)					

^a^Differences were considered significant at *P*<.05.

^b^HO/MO/SMO: house officer/medical officer/senior medical officer.

^c^Differences were considered significant at *P*<.01.

Logistic regression analysis showed that respondents working in tertiary health care facilities were 80% less likely (adjusted odds ratio [AOR] 0.20, 95% CI 0.07-0.59; *P*=.003) to have adequate BLS knowledge and skills compared to those in primary health care facilities. Respondents in the Volta and Oti regions were 4.94 times more likely (AOR 4.94, 95% CI 1.17-20.80; *P*=.03) to possess adequate BLS knowledge and skills compared to those in the Bono East region (Table 2).

**Table 2:** Logistic regression analysis of the factors associated with respondents’ basic life support knowledge and skill scores.

Variable			UOR^a^ (95% CI)		*P* value	AOR^b^ (95% CI)		*P* value
**Profession**								
	Physician assistant/nurse	1.67 (0.89-3.14)		.11		1.45 (0.70-2.96)	.32	
	House officer/medical officer/senior medical officer	0.41 (0.13-1.27)		.12		0.47 (0.14-1.58)	.22	
	Specialist/trainee specialist	Ref^c^		Ref		Ref	Ref	
**Facility**								
	Primary	Ref		Ref		Ref	Ref	
	Secondary	1.00 (0.50-2.02)		>.99		0.97 (0.44-2.12)	.94	
	Tertiary	0.26 (0.10-0.71)		.009		0.20 (0.07-0.59)	.003^d^	
**Region**								
	Ashanti	1.91 (0.54-6.82)		.32		1.16 (0.30-4.49)	.83	
	Bono East	Ref		Ref		Ref	Ref	
	Central	1.90 (0.51-7.08)		.34		1.48 (0.37-5.97)	.58	
	Eastern	2.63 (0.72-9.61)		.15		1.29 (0.30-5.50)	.73	
	Greater Accra	1.35 (0.43-4.26)		.61		0.95 (0.27-3.36)	.93	
	Northern	2.35 (0.69-7.96)		.17		1.48 (0.38-5.82)	.58	
	Volta/Oti	6.50 (1.90-22.23)		.003		4.94 (1.17-20.80)	.03^e^	

^a^UOR: unadjusted odds ratio.

^b^AOR: adjusted odds ratio.

^c^Ref: reference category.

^d^Significant at *P*<.01.

^e^Significant at *P*<.05.

### ACLS Training

In total, 124 health care professionals received ACLS training, and standardized questionnaires were administered for the assessment of knowledge and skills. Most (73/124, 58.8%) were males. The majority (89/124, 71.7%) were doctors (house officer/medical officer/senior medical officer). More than half (64/124, 51.6%) worked in tertiary health care facilities. Most (55/124, 44.3%) of the ACLS trainings took place in the Greater Accra region. The majority (91/124, 73.4%) showed adequate knowledge and skills in ACLS. Statistically significant differences in ACLS knowledge and skills scores were observed based on gender, profession, and facility (*P*<.05; see Table 3).

**Table 3:** Sociodemographic characteristics of the respondents receiving advanced cardiac life support training.

Variable			Total (n=124), n (%)		Advanced cardiac life support knowledge score, n (%)					*P* value	
					Adequate (n=91)		Inadequate (n=33)				
**Gender**											<.001^a^
	Female	51 (41.1)		26 (28.6)		25 (75.8)					
	Male	73 (58.9)		65 (71.4)		8 (24.2)					
**Profession**											.003^b^
	Physician assistant/nurse	4 (3.2)		0 (0)		4 (12.1)					
	House officer/medical officer/senior medical officer	89 (71.8)		68 (74.7)		21 (63.6)					
	Specialist/trainee specialist	31 (25)		23 (25.3)		8 (24.2)					
**Facility**											.045^c^
	Primary	20 (16.1)		12 (13.2)		8 (16.1)					
	Secondary	40 (32.3)		26 (28.6)		40 (32.3)					
	Tertiary	64 (51.6)		53 (58.2)		64 (51.6)					
**Region**											.34
	Ashanti	12 (9.7)		10 (11)		2 (6.1)					
	Bono East	11 (8.9)		8 (8.8)		3 (9.1)					
	Central	11 (8.9)		5 (5.5)		6 (18.2)					
	Eastern	11 (8.9)		9 (9.9)		2 (6.1)					
	Greater Accra	55 (44.4)		39 (42.9)		16 (48.5)					
	Northern	12 (9.7)		10 (11)		2 (6.1)					
	Volta/Oti	12 (9.7)		10 (11)		2 (6.1)					

^a^Differences were considered significant at *P*<.001.

^b^Differences were considered significant at *P*<.01.

^c^Differences were considered significant at *P*<.05.

Compared to their female counterparts, males were 7.05 times more likely (AOR 7.05, 95% CI 2.69-18.46; *P*<.001) to have adequate knowledge and skills in ACLS (see Table 4).

**Table 4:** Logistic regression analysis of the factors associated with respondents’ advanced cardiac life support knowledge and skill scores.

Variable		UOR^a^ (95% CI)	*P* value	AOR^b^ (95% CI)	*P* value
**Gender**					
	Female	Ref^c^	Ref	Ref	Ref
	Male	7.81 (3.12-19.54)	<.001	7.05 (2.69-18.46)	<.001^d^
**Profession**					
	Physician assistant/nurse	—^e^	—	—	—
	House officer/medical officer/senior medical officer	0.89 (0.35-2.28)	.80	0.66 (0.22-1.98)	.46
	Specialist/trainee specialist	Ref	Ref	Ref	Ref
**Facility**					
	Primary	3.21 (1.06-9.70)	.04	2.96 (0.79-11.13)	.11
	Secondary	2.59 (1.04-6.50)	.04	2.25 (0.80-6.30)	.12
	Tertiary	Ref	Ref	Ref	Ref

^a^UOR: unadjusted odds ratio.

^b^AOR: adjusted odds ratio.

^c^Ref: reference category.

^d^Significant at *P*<.001.

^e^Not applicable.

## Discussion

### Principal Findings

This study aims to evaluate the knowledge and skills of health care professionals immediately after training in Ghana on managing cardiac arrest through BLS and ACLS. We found that 75% of the health care professionals had adequate knowledge and skills in BLS, which was notably higher than that reported in other studies [2,9,11,12]. In similar training contexts, these percentages were 12% in Nepal [9] and 54% in Yemen [12]. Our results differ from studies in Afghanistan [2] and Pakistan [11], where 5% and 42% of respondents, respectively, had adequate BLS knowledge. The differences across these studies could be attributed to variations in research methods, the cutoff points used for determining knowledge levels, the effectiveness of the training programs, and whether assessments were conducted immediately after training. Additionally, prior BLS training may account for these variations, as Chaudhary et al [9] reported a strong correlation between knowledge scores and previous training.

This study reveals significant associations between BLS knowledge and skill scores and profession, facility, and region; however, no research has shown an association between the knowledge and skill scores of professions and facility type or region for comparison. We found that health care professionals working in tertiary health care facilities were less likely to have adequate knowledge and skills about BLS than those working in primary health care facilities. The reason could be that primary health care facilities in Ghana often provide basic emergency care as part of their primary services to communities; therefore, they receive more focused and frequent training on BLS techniques as part of their routine responsibilities compared to tertiary health care facilities, which prioritize specialized care for severe medical conditions, resulting in less emphasis on BLS training. The disparity in the findings could also be attributed to the assumption of prior knowledge and skills from practicing in a tertiary health facility.

Our study shows that health care professionals in facilities within the Volta and Oti regions possessed more adequate knowledge and skills in BLS compared to those in the Bono East region. This may be due to differences in the frequency of BLS training programs conducted in these areas. Facilities in the Volta and Oti regions might have more regular BLS training sessions than those in Bono East. Additionally, regions with better access to resources may facilitate more effective training for health care professionals, resulting in higher retention of BLS knowledge and skills; however, there is no evidence in the literature to support this justification.

Regarding ACLS, this study shows that 73% of the participants possessed adequate knowledge and skills, which contrasts with that reported in studies conducted in Ethiopia [1] and Spain [4]. Those studies showed that the majority, at 40% and 28%, respectively, had adequate knowledge of ACLS [1,4]. The difference may relate to variations in study methodologies, how knowledge was classified, and the previous training received on ACLS; none of these assessments were based on AHA-certified ACLS training. Adal and Emishaw [1] revealed that health care professionals who had previously received ACLS training were 5 times more knowledgeable than those who had not.

We found a significant difference between the ACLS knowledge and skill scores and gender. Compared with their female counterparts, male health care professionals were more likely to have adequate knowledge and skills in ACLS. This may be because our study had more male participants than female counterparts. Another reason could be the differences in the types or frequency of the clinical experiences.

### Strengths and Limitations

This study’s strength is that it is the first in Ghana to evaluate health care professionals' knowledge and skills in managing cardiac arrest by using BLS and ACLS. It also included professionals from various backgrounds, facility types, and regions, thereby providing a thorough assessment across different parts of Ghana. In addition, our study created a standard for BLS and ACLS training and assessment that could be used in Ghana and other countries to support and monitor health care professionals’ proficiency.

However, our study has some limitations. First, the evaluation of BLS and ACLS knowledge and skills was based on posttraining tests, which may not accurately reflect participants’ real-world performance in emergencies and could introduce social desirability bias. Second, participants were recruited from only 8 regions and may not represent all the health care professionals in Ghana, potentially leading to selection bias. Third, there are no data on the specific deficiencies encountered.

### Conclusion

Given an opportunity to learn and practice, health care professionals in Ghana attain adequate knowledge and skills in BLS and ACLS. BLS knowledge and skill scores were significantly associated with profession, facility, and region. Additionally, ACLS knowledge and skill scores were found to be associated with gender, profession, and facility. It is recommended that BLS and ACLS training be provided regularly to all health care professionals across all regions to improve their ability to respond effectively to medical emergencies.

## Abbreviations

ACLS: advanced cardiac life support
AHA: American Heart Association
AOR: adjusted odds ratio
BLS: basic life support
CPR: cardiopulmonary resuscitation
ECC: emergency cardiovascular care

## Data Availability

The data supporting the study's findings are available from the corresponding author upon request.
